# Developing Entrustable Professional Activities for Chemical Pathology registrars in South Africa

**DOI:** 10.4102/jcmsa.v3i1.133

**Published:** 2025-02-14

**Authors:** Rivak Punchoo, Tahir S. Pillay

**Affiliations:** 1 Division of Chemical Pathology, Department of Pathology, Faculty of Health Sciences, University of Cape Town, Cape Town, South Africa; 2 Department of Chemical Pathology, National Health Laboratory Service, Cape Town, South Africa; 3 Department of Chemical Pathology, Faculty of Health Sciences, University of Pretoria, Pretoria, South Africa; 4 Department of Chemical Pathology, National Health Laboratory Service, Pretoria, South Africa

**Keywords:** EPA, Entrustable Professional Activities, workplace-based training, Chemical Pathology, laboratory medicine, constructive alignment, validation of EPAs, standardisation of learning opportunities

## Abstract

The College of Medicine South Africa (CMSA) has embarked on developing Entrustable Professional Activities (EPAs) for workplace-based training. There is currently limited guidance for developing EPAs in Chemical Pathology. This report explores factors that impact EPA development in Chemical Pathology, derived from health education theory and evidence, as well as relevant contextual variables impacting registrar training in South Africa. We discuss four factors to consider during the elaboration of EPAs for South African registrars in Chemical Pathology to enhance fitness for workplace-based assessment. Firstly, EPAs require the application of constructive alignment to link the national syllabus, core competencies and milestones, training methods, and formative assessment strategies during the trainee learning trajectory. Secondly, the elaboration of a complete EPA is assisted by utilising a standardised template with defined sectional headings and contents structured for laboratory medicine. Thirdly, quality assurance of draft EPAs requires validation to improve curriculum alignment, and facilitate EPA revision. Finally, a critical emergent issue for Chemical Pathology workplace-based training is the development of standardised learning opportunities for operationalising a national EPA programme.

**Contribution:**

Charting the early steps in developing EPAs for training Chemical Pathology registrars in South Africa identifies the application of the educational tenet of constructive alignment, utilising a standardised template for EPA design, validating draft EPAs and creating equitable learning opportunities for all trainees.

## Introduction

The training of South African registrars in Chemical Pathology is conducted at universities linked to academic hospitals and National Health Laboratory Service (NHLS) platforms. The registrars are formally taught by pathologist consultants at each institution with ad hoc external training opportunities, such as national NHLS pathology conferences and online NHLS webinar programmes. Registrars can also access training support from private laboratory pathologists and scientists for day visits to explore novel test methodologies, specialist testing and conduct case-based tutorials – although, these practices show inter-institutional variability. Many NHLS platforms also accommodate scientists who contribute to teaching, especially, scientific methodology for specialist tests and wet-bench practicals. Registrars also interact with medical technologists and technicians in their daily work to resolve laboratory problems and support operational delivery. While registrars write primary and exit specialist examinations, current training does not evaluate performance of registrars on workplace-based tasks and provide feedback to support their learning on the job.

Entrustable Professional Activities (EPAs) define discrete professional units of work that are independently executable and measurable, and operationalise medical competencies for trainees in the healthcare environment. Entrustable Professional Activities allow assessors to make entrustment decisions about a trainee’s performance on essential tasks.^1,2^

The College of Medicine South Africa (CMSA) has embarked on introducing EPAs for national post-graduate specialist training. The introduction of EPAs will contribute to formative assessment for trainees to enable entrustment decisions for unsupervised work practice and provide multiple feedback opportunities to enhance learning.

The development of EPAs for registrar training in Chemical Pathology in South Africa highlights the need to consider essential educational principles and evidence to construct a set of work activities that span the post-graduate curriculum. Currently, there is no clear framework to guide EPA formulation in Chemical Pathology.^3^

This brief practice report highlights four factors that can assist the early steps in EPA design for Chemical Pathology. These factors are constructive alignment within a national curriculum to develop core EPAs, elaborating EPAs by a standardised template, validating EPAs for fitness of purpose, and developing accessible and fair training opportunities for registrars.

## Constructive alignment in Entrustable Professional Activities development

Constructive alignment forges an explicit linkage between learning outcomes articulated in the syllabus, teaching methodologies, and assessment approaches measured against an acceptable standard.^4^ The task selection for an EPA can be guided by essential everyday professional work activities, high-risk or error-prone tasks, or tasks that function as exemplars for competency – all EPAs then can be mapped to the curriculum and connected with entrustment milestones.^1,5^ In a scoping review to identify the rationalisation and selection of EPAs, Hennus et al. identified three dominant logics that guide EPA development in health sciences: service provision, procedures, and disease or patient categories.^6^ These categories are helpful to Chemical Pathology in defining essential professional work units from the national syllabus, which holistically encompasses daily professional practice. These categories also assist the alignment of EPAs to specified learning objectives, competency domains, and relevant knowledge, skills, and attitude categories stated or alluded to in the registrar’s national curriculum.

**FIGURE 1:**
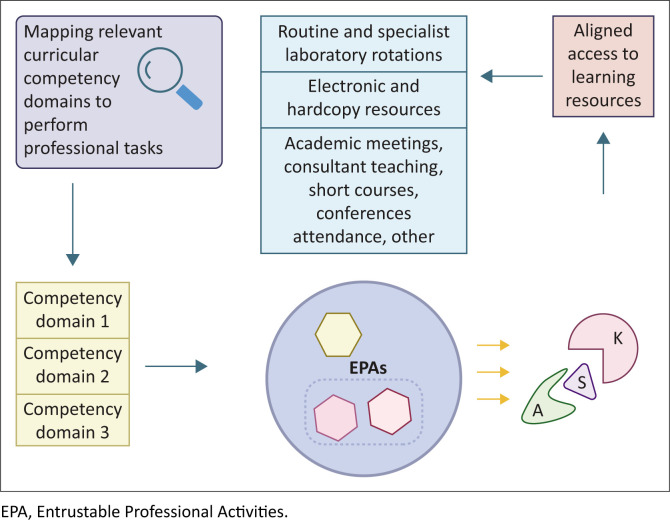
Essential steps in developing Entrustable Professional Activities for Chemical Pathology (created with BioRender.com).

Constructive alignment facilitates the integration of relevant competencies within each EPA, emphasising a synthetic approach to operationalising competencies.^7^ Further, it identifies linkage to relevant learning opportunities available to trainees to learn and practise EPAs. Figure 1 summarises the core elements of constructive alignment for EPA development in Chemical Pathology in South Africa.

One or more competency domains derived from the national curriculum are formulated and mapped to each EPA. Therefore, the competency domains selected are specific for each EPA and describe the core qualities necessary to perform the task. Nested and stand-alone EPAs (shown within the blue circle) are then formulated to cover all essential professional tasks spanning the profession. The ‘nested’ EPAs are within the rectangle shape circumscribed by a dashed line. The yellow hexagon indicates a stand-alone EPA. The cognitive, psychomotor, and affective dimensions required for each EPA are indicated by the abbreviations K (Knowledge), S (Skills) and A (Attitudes). The resources that are aligned to achieve learning are labelled in the blue stacked rectangle block.

## Elaborating Entrustable Professional Activities by adopting a standardised template

The writing of EPAs is challenging and requires a content expert panel and guidance from persons trained formally in health education to ensure respective contributions of sound subject matter expertise and the application of pedagogic tenets in developing EPAs. A standardised template is available to write up generic EPAs (Table 1),^2,5,8,9^ and the sectional headings expand a fully detailed mini-curriculum that enables the professional task to be performed.^5^ A well-elaborated EPA has a clear goal to prepare learners and assessors for entrustment decisions. Table 1 provides further guidance on how to expand each section when writing Chemical Pathology EPAs.

**TABLE 1:** A template of sectional headers, and guidelines to elaborate content areas for a full Entrustable Professional Activities description.^5,10^

Sectional heading	Guidelines to formulate the contents of each section
1. Title	EPA titles usually consist of ±10 words clearly describing the activity, for example, ‘Evaluating patients’ biochemical results for the diagnosis of disease’. The EPA title should utilise an appropriate verb without adjectives or adverbs that focus on proficiency. The title should consider appropriate laboratory diction and clearly communicate to the registrar and assessor the activity undertaken by the EPA in the context of the laboratory or clinical environment.
2. Description of the EPA: Specification and limitation	The precise activity should be specified in this section, which considers all practical aspects of the professional job unit. Nested EPAs can be inserted in the umbrella EPA to provide more minor activities which consider trainee level, and progression through the training programme and achievement of milestones. The limitations provide clear articulation of professional activity not included in the EPA and thus define the boundary of the EPA. Trainees and assessors should be clear on the contents of the EPA and understand what activity needs to be completed in the EPA.
3. Competency domains	Each EPA states the relevant competency domain or domains applicable to the EPA. The canadian medical education directions for specialists (CanMEDS) competency framework can be used in constructing EPAs for Chemical Pathology. Competency domains can be mapped to the national registrar curriculum and recommendations by the National Health Laboratory Service Chemistry Expert Committee. The competency domains selected should correspond with the Description section of the EPA (Sectional heading 2). A competency matrix covering the complete set of EPAs can be created to provide a table of core competencies selected for each EPA, which is mapped across the registrar training curriculum and provides a vertical map of the EPA curriculum.
4. Knowledge, skills and attitudes (KSA), and relevant experience	All KSA domains to perform the EPA are stated in this section and linked to the specifications of the EPA and the core competency sections for Chemical Pathology (Sectional heading 3). This section should comprehensively state all necessary KSA domains that require engagement to perform the Chemical Pathology task safely and proficiently.
5. Resources and learning opportunities	This section has been added to the standard template to accommodate the heterogeneity in resource infrastructure in laboratory resources (specialist training opportunities, access to teaching infrastructure and access to consultant teachers/assessors). It foregrounds the identification of standardised resources across institutions to ensure registrars have access to the necessary training materials and learning opportunities. This section should explicitly state resources linked to KSA domains (Section heading 4) to facilitate the registrar perform all aspects of the professional task to achieve assigned competencies in the laboratory and clinical workplace.
6. Assessment	A careful selection and integration of relevant tools to measure registrar performance is required to support assessment objectively and the entrustment decision taken. This can consider direct observation or indirect methods for laboratory medicine such as review of artefacts, interrogating reasoning by case discussion and multisource feedback. More than one tool can be selected to comprehensively measure registrar performance and triangulate registrar performance data to make sound *ad hoc* formative and longitudinal summative entrustment decisions, if needed.
7. Level of entrustment	Formative assessment is framed as entrustment for registrars to undertake essential activities by an entrustment level of supervision. A five-level scale of entrustment and supervision is awarded based on registrar’s performance for each EPA submission: Not allowed to practise EPA. Allowed to practise the EPA only under proactive, full supervision. Allowed to practise EPA only under reactive/on-demand supervision. Allowed to practise the EPA unsupervised. Allowed to supervise others in the practice of EPA. The assessor’s entrustment decisions should consider the registrar training level, and milestones that will be achieved at different time points along the training trajectory during the registrarship. Areas for improvement need to be communicated to trainees via narrative feedback to enable the development of feasible plans to enhance learning at each formative EPA assessment.
8. Time to expiration	This section is optional because the CMSA and HPCSA do not mandate a policy for renewal of skillsets after completion and passing of the registrarship. Within registrarship training, this section should consider the erosion of performance ability, and the time interval required for the repetition of the EPA and frequency of assessments required to sustain optimal EPA performance during the duration of the training programme.
9. Potential risks	This section considers egregious outcomes for the failed EPA performance. The impact on clinical and laboratory medicine areas needs to be holistically reviewed and weighted to evaluate clinical, financial, ethical and governance factors on the Chemical Pathology laboratory and the broader clinical learning environment.

CanMEDS, Canadian Medical Education Directions for Specialists; CMSA, College of Medicine South Africa; EPA, Entrustable Professional Activities; HPCSA, Health Professionals Council of South Africa.

A basic example of an EPA is provided in Box 1 that applies guiding points listed in Table 1 to a real-world professional task in the Chemical Pathology laboratory.

BOX 1 Example of an authentic task in the Chemical Pathology laboratory written as an Entrustable Professional Activity.

1. **Title: Solving quality control problems in the Chemical Pathology laboratory**
2. **Specification and limitation**
  **Specification**
  - This EPA will evaluate your ability to analyse and troubleshoot internal and external quality control (QC) problems in the Chemical Pathology laboratory for routine tests.
  - A systematic root cause analysis will need to be conducted, and an intervention plan to correct and prevent the quality control problem will need to be developed and implemented.
  **Limitation**
  - The effective implementation of the QC intervention is partially dependent on multiple stakeholders involved in the daily laboratory management and may not reflect registrar autonomous decision-making or resolution skillsets.
3. **Domains of competence**
  - Medical expert
  - Communicator
  - Collaborator
  - Manager/Leader
4. **Knowledge, Skills and Attitudes (KSA) and Learning Opportunities and Resources**
  - **Knowledge:** Root cause analysis and management implementation tools such as the PDCA cycle; and basic knowledge of interpreting QC chart data using standardised tools and essential laboratory statistics
  - **Skills:** Graphing skills, data analysis and interpretation, and writing of interventional reports to support laboratory accreditation
  - **Attitudes:** Communication skills to accommodate multiple laboratory stakeholder interests, and coping with personal emotional responsiveness when dealing with difficult stakeholders
5. **Learning opportunities and resources**
  **5.1. Learning opportunities**
  - Review of internal QC alerts and failures, and EQA performance data for routine tests
  **5.2. Learning resources**
  - Academic meetings discussing laboratory management principles at institutional and national levels
  - Prescribed textbooks and literature by CMSA for understanding QC in the laboratory and implementing QC improvement in the laboratory
6. **Assessment evidence to inform entrustment decisions**
  **6.1 Assessment format**
  - Case-based discussion
  **6.2 Number of observations/year; total observations**
  - Four EPA submissions per year covering multiple areas and of varying level of complexity along the total testing process
7. **Entrustment level of supervision assigned to the stage of training**
  **Year 1:** Level of entrustment: direct supervision
  **Year 2:** Level of entrustment: direct supervision or partial entrustment
  **Year 3:** Level of entrustment: partial/full entrustment
  **Year 4:** Level: unsupervised (full entrustment decision)
8. **Expiration date**
  - Annual re-evaluation over the 4-year registrarship
9. **Potential risks of failure**
  - Waste of laboratory consumables and staff-time resource
  - Validation of false results that endanger patient safety

EPA, Entrustable Professional Activities; CMSA, College of Medicine South Africa; EQA, external quality assessment; PDCA, plan-do-check-act.

## Validation of Entrustable Professional Activities improves structure, quality and implementation

Validating draft EPAs nationally will ensure the Chemical Pathology EPAs are relevant, aligned with purpose, span discrete and essential professional work, and encourage revisions to achieve a consensus list by stakeholders. Various strategies are described to validate EPAs. These include expert meetings, surveys and interviews, the Delphi procedure, the nominal group technique, and EPA evaluation rubric tools.^1,11^ The NHLS Chemistry Expert Committee is a valuable resource for developing EPAs among Chemical Pathology experts and iteratively reviewing drafts to achieve a consolidated EPA list. The engagement of local experts also encourages commitment to finalising EPA development and implementation.

## Standardising learning opportunities across national registrar training sites

The active construction of learning opportunities for all registrars during all training phases will enable the standardisation of learning between national training sites. Standardisation of learning opportunities is important in South Africa as university resource variation reflects historical geo-political inequities in resource allocation. Additionally, pathology test menus show regional variation between the training state laboratory academic hospitals, such as variation in specialist test menus.

Initiatives to equalise training opportunities for Chemical Pathology should consider the development of feasible interventions and further integration with current training opportunities supported by the National Health Laboratory Service (NHLS) platform. Examples of potential learning opportunities are highlighted in Table 2. These training resources and opportunities need to be built into the broader EPA curriculum, which explicitly states the essential resources required for training each EPA.

**TABLE 2:** Development of national training opportunities to support essential Knowledge, Skills and Attitudes domains articulated in Entrustable Professional Activities.

Development of essential knowledge, skills and attitudes to support registrar EPA training	Training opportunity	Commentary
Laboratory skills	Short laboratory rotations at private laboratories and within the National Health Laboratory Service	Registrar access and training to instrumentation and methodologies should be harmonised across regional training sites. The logistics to set up rotations and develop an interface with private laboratories require careful planning and integration with the national EPA programme.
	Participation in workshops that target the development of practical skills essential for Chemical Pathology trainees	The NHLS currently supports works skills development of registrars, and integration within EPA frameworks needs to be considered by training centres. External workshops offered by private diagnostic services and laboratories will also need to be sourced where training opportunities limit registrar training and practice of EPAs. Workshop opportunities offered by the national societies such as the SAACB (South African Association for Clinical Biochemistry and Laboratory Medicine) should be taken advantage of.
Essential knowledge areas mapped in EPAs	Registrars can attend online national academic meetings	The current ECHO™ talks by the NHLS support online training. These talks and online resource materials should be integrated into EPA design to support national EPA rollout in Chemical Pathology.
	Attendance at national and international conferences in laboratory medicine	Essential laboratory conferences need to be considered to maximise learning opportunities covering broad and current topics in Chemical Pathology. The NHLS does support registrars attending national and internal conferences each year, and training institutes should curate attendance to align with EPAs. The prohibitive costs of conference attendance require careful planning by regional sites and further integration within the national EPA programme.
Development of attitudes and professional competencies relevant to professional pathologist practice	Attendance of workshops to support the development of professional areas that include conflict management and adherence to sound ethical practice in laboratory medicine	Online and in-person workshops need to be sourced which are affordable to registrars. Again a national society such as the SAACB should be approached to facilitate. Training opportunities of ‘soft skill’ sets should not be neglected as these skills are crucial to facilitate the professional development of a pathologist. Learning opportunities should align with Section 4 of the EPA, which comprehensively describes relevant attitudes and experiences required by trainees for each EPA.

EPA, Entrustable Professional Activities; NHLS, National Health Laboratory Service.

The shortage of pathologists at some training sites also requires redress by NHLS and universities to support the daily training needs of registrars at regional training sites. Notably, adequate consultant availability and faculty development to conduct regular EPA assessments are crucial for valid and reliable assessments and registrars’ formative learning.^12^

## Conclusion

The initial steps to construct EPAs in Chemical Pathology for South African registrars should be informed by aligning the national syllabus with a core competency framework, aligning learning opportunities, and selecting appropriate workplace-based assessment (WBA) tools for formative assessment. Entrustable Professional Activities can be elaborated using a standardised template, and education tools and expert panels can validate the draft versions to ensure fitness for purpose. During the development of EPAs in Chemical Pathology, fair access to learning opportunities should be cultivated for the national EPA programme rollout.
